# Urinary epidermal growth factor/monocyte chemotactic peptide 1 ratio as non-invasive predictor of Mayo clinic imaging classes in autosomal dominant polycystic kidney disease

**DOI:** 10.1007/s40620-022-01468-w

**Published:** 2022-11-07

**Authors:** Maria Teresa Rocchetti, Francesco Pesce, Silvia Matino, Giovanni Piscopo, Ighli di Bari, Francesco Trepiccione, Giovanna Capolongo, Maria Antonietta Perniola, Xuewen Song, Saima Khowaja, Amirreza Haghighi, Dorien Peters, Simona Paolicelli, Paola Pontrelli, Giuseppe Stefano Netti, Elena Ranieri, Giovambattista Capasso, Marco Moschetta, York Pei, Loreto Gesualdo, (Studio PRE. MED. (MEDicina di PREcisione) Prog.n.F/050065/01-02/X32)

**Affiliations:** 1 Department of Clinical and Experimental Medicine, University of Foggia, 71122 Foggia, Italy; 2 Department of Emergency and Organ Transplantation, Nephrology, Dialysis and Transplantation Unit, University of Bari “Aldo Moro”, Bari, Italy; 3 Nefrologia, Dipartimento di Scienze Mediche Traslazionali, Università della Campania “Luigi Vanvitelli”, Naples, Italy; 4 Nefrologia e Dialisi, Presidio Ospedaliero Valle d’Itria, Martina Franca, Taranto, Italy; 5 Division of Nephrology, University Health Network and University of Toronto, Toronto, ON Canada; 6 Department of Human Genetics, Leiden University Medical Center, Leiden, The Netherlands; 7 Department of Medical and Surgical Sciences, Clinical Pathology Unit, Center of Molecular Medicine, University of Foggia, 71122 Foggia, Italy; 8 Biogem Research Institute, Ariano Irpino, Italy

**Keywords:** ADPKD, EGF/MCP1, Risk prediction, CKD progression urine biomarkers

## Abstract

**Background:**

Age- and height-adjusted total kidney volume is currently considered the best prognosticator in patients with autosomal dominant polycystic kidney disease. We tested the ratio of urinary epidermal growth factor and monocyte chemotactic peptide 1 for the prediction of the Mayo Clinic Imaging Classes.

**Methods:**

Urinary epidermal growth factor and monocyte chemotactic peptide 1 levels were measured in two independent cohorts (discovery, *n* = 74 and validation set, *n* = 177) and healthy controls (*n* = 59) by immunological assay. Magnetic resonance imaging parameters were used for total kidney volume calculation and the Mayo Clinic Imaging Classification defined slow (1A–1B) and fast progressors (1C–1E). Microarray and quantitative gene expression analysis were used to test epidermal growth factor and monocyte chemotactic peptide 1 gene expression.

**Results:**

Baseline ratio of urinary epidermal growth factor and monocyte chemotactic peptide 1 correlated with total kidney volume adjusted for height (*r* = − 0.6, *p* < 0.001), estimated glomerular filtration rate (*r* = 0.69 *p* < 0.001), discriminated between Mayo Clinic Imaging Classes (*p* < 0.001), and predicted the variation of estimated glomerular filtration rate at 10 years (*r* = − 0.51, *p* < 0.001). Conditional Inference Trees identified cut-off levels of the ratio of urinary epidermal growth factor and monocyte chemotactic peptide 1 for slow and fast progressors at > 132 (100% slow) and < 25.76 (89% and 86% fast, according to age), with 94% sensitivity and 66% specificity (*p* = 6.51E−16). Further, the ratio of urinary epidermal growth factor and monocyte chemotactic peptide 1 at baseline showed a positive correlation (*p* = 0.006, *r* = 0.36) with renal outcome (delta-estimated glomerular filtration rate per year, over a mean follow-up of 4.2 ± 1.2 years). Changes in the urinary epidermal growth factor and monocyte chemotactic peptide 1 were mirrored by gene expression levels in both human kidney cysts (epidermal growth factor: − 5.6-fold, fdr = 0.001; monocyte chemotactic peptide 1: 3.1-fold, fdr = 0.03) and *Pkd1* knock-out mouse kidney (*Egf*: − 14.8-fold, fdr = 2.37E-20, *Mcp1*: 2.8-fold, fdr = 6.82E−15).

**Conclusion:**

The ratio of urinary epidermal growth factor and monocyte chemotactic peptide 1 is a non-invasive pathophysiological biomarker that can be used for clinical risk stratification in autosomal dominant polycystic kidney disease.

**Supplementary Information:**

The online version contains supplementary material available at 10.1007/s40620-022-01468-w.

## Introduction

Autosomal dominant polycystic kidney disease (ADPKD) is the most common inherited kidney disorder worldwide and accounts for 5–8% of end-stage renal disease (ESRD) [1]. It is typically characterized by the age-dependent development of innumerable cysts with bilateral kidney enlargement which eventually leads to advanced kidney failure in a majority of patients. Cyst enlargement leads to expansion of total kidney volume (TKV) at a quasi-exponential rate (i.e. on average, 5% per year) during adult life in patients with ADPKD, while their kidney function typically remains stable for 3–4 decades [1]. Large variability of disease severity in ADPKD has been well-documented [1, 2] and is likely due to a complex gene-environment interaction [3]. Observational studies have shown that age, male sex, hypertension, truncating *PKD1* mutations, and TKV are associated with an increased risk of chronic kidney disease (CKD) progression [4]. Using age- and height-adjusted TKV, the Mayo Clinic Imaging Classification provides a validated tool for risk prediction in ADPKD and stratifies cases into five prognostic classes (1A–1E) with increasing rates of estimated glomerular filtration rate (eGFR) decline [5]. However, access to magnetic resonance imaging, the need for special radiology support, and costs may limit the widespread use of this approach in the clinical setting. There is an urgent need for simpler and well-validated biomarkers to identify “high-risk” patients for novel disease-modifying treatment, such as Tolvaptan.

The pathophysiology of ADPKD is not fully understood [6]. However, recent studies have documented that kidney inflammation can promote progression of experimental ADPKD [7, 8]. In this context, epidermal growth factor (EGF) is a kidney-specific protective cytokine [9] with key functions in cell differentiation and regeneration, and its tissue expression is down-regulated in human *PKD1* cysts [10]. By contrast, monocyte chemoattractant peptide 1 (MCP1), an inflammatory chemokine, displays increased expression in cystic tissues of *Pkd1* knock-out mice and human *PKD1* kidneys [10, 11]. Tubular genetic ablation of *Mcp1* was associated with reduced cystic disease severity in a *Pkd1* knock-out mouse model [8]. Thus, both EGF and MCP1 are strong candidate biomarkers for predicting the progression of renal damage in ADPKD. Urinary EGF and MCP1 levels have already been individually associated with increased risk of CKD progression in ADPKD patients [12–14], while the ratio of these two cytokines (uEGF/MCP1) has proven to be a useful prognostic biomarker in IgA nephropathy [15]. Here, we tested uEGF/MCP1 as a non-invasive clinical biomarker for progression in ADPKD.

## Materials and methods

### Study design

From January 2018 to November 2018, 74 ADPKD patients from the Nephrology Unit of the University of Bari, Martina Franca Hospital and the University of Campania “Luigi Vanvitelli” were recruited in a prospective cohort study [PRE.MED.]

The ADPKD validation cohort was recruited from May 2018 to January 2019 at the Centre for Innovative Management of Polycystic Kidney Disease, University Health Network, Toronto, Ontario, Canada, with informed consent approved by the local Ethical Committee. The diagnosis of APDKD was made based upon the revised Pei-Ravine criteria. The exclusion criteria were: age < 18 years, recent surgery (< 30 days), current myocardial infarction (< 30 days), active infections (HBV, HCV, HIV), current or previous solid or haematologic malignancies (< 1 year), inflammatory or systemic granulomatous diseases, diabetes mellitus, suspicion of other nephropathy superimposed on ADPKD, solid or bone marrow transplantation, current or previous steroid therapy (< 30 days), metformin therapy, previous tuberculosis, pregnancy or recent delivery, dialysis.

At baseline visit, we recorded weight, height and clinical data and collected blood and urine samples from each patient when the magnetic resonance imaging study was performed. Five patients of the first cohort did not undergo magnetic resonance imaging due to contraindications not related to ADPKD. TKV was measured using the ellipsoid equation and adjusted for height; eGFR (mL/min/1.73 m^2^) was measured using the Chronic Kidney Disease EPIdemiology equation; creatininuria, proteinuria, EGF and MCP1 were measured on the second morning urine sample. After a median follow-up of 4.2 ± 1.2 years, eGFR was measured in 59 discovery cohort patients and clinical and genetic data were collected. The Predicting Renal Outcome in Polycystic Kidney Disease (PROPKD) score of 59 patients was calculated [30] (Table 1).

**Table 1:** Demographic, clinical and laboratory characteristics of the discovery and validation cohorts of patients with ADPKD

	Discovery	Validation	Healthy
No. of patients	74	177	59
Sex (F/M)	44/30	92/85	33/26
Age (years)	44.86 ± 12.67	44.90 ± 13.64	41.71 ± 7.37
Serum creatinine (mg/dl)	1.39 ± 0.76	1.10 ± 0.56	0.78 ± 0.23
eGFR (ml/min/1.73 m^2^)	67.0 ± 32.0	79.4 ± 25.4	94.8 ± 10.1
Genetics (nr. Patients)			
Truncating PKD1	29		
Nontruncating PKD1	12	–	–
PKD2	18		
No data	15		
PROPKD score risk classes:			
Low	23	–	–
Intermediate	25		
High	11		
Missing	15		
Delta-eGFR/year (ml/min/1.73 m^2^)/years	− 20 (− 29 to − 6)	–	–
TKV (ml)	1849 (350–7373)	1267 (243–5047)	
Ht-TKV (ml)	1161 (201–4256)	738 (146–3027)	
uEGF (pg/mgCr)	14.185 ± 11,265	18,628 ± 9098	40,011 ± 26,208
uMCP1(pg/mgCr)	446.4 ± 374.8	427.3 ± 446.6	163.9 ± 183.7
uEGF/MCP1	38.4 (11.3–90.4)	53.2 (26.4–106.6)	282.1 (159.6–511.8)
Mayo Class (no.)			
1A	6	30	
1B	11	56	
1C	16	47	
1D	20	32	
1E	9	12	
Atypical	1 (*11 unclassified)	0	

The Mayo Clinic Imaging Classification for ADPKD defines five classes of patients according to prognosis: classes 1A–1B and 1C–1E were defined as slow and fast progressors, respectively.

### Cytokine measurements

uMCP1 and uEGF measurements were performed using ELISA kits (Quantikine ELISA Immunoassay, R&D systems, Minneapolis, USA) according to the manufacturer’s instructions.

### Microarray experiments

Human *EGF* and *MCP1* expression data were retrieved from a previous study we published on global gene profiling on cysts of different size (*n* = 13) and minimally cystic tissue (MCT, *n* = 5) from five *PKD1* human polycystic kidneys using Affymetrix HG-U133 Plus 2.0 arrays [10]. Mouse *Egf* and *Mcp1* expression data were retrieved from a previous study we published on global gene profiling on *Pkd1* knock-out (*n* = 12), and wild type (*n* = 10) kidneys using Affymetrix GeneChip Mouse Gene 2.0 ST Arrays [16].

### Microarray validation by qRT-PCR

Microarray validation was performed in a set of cysts (*n* = 38: small cysts, SC = 16; medium cysts, MC = 19; large cysts, LC = 3) and minimally cystic tissue (MCT; *n* = 14) samples derived from human *PKD1* kidneys, as well as in normal renal cortical samples (*n* = 4) and animal tissues. In addition, a set of *Pkd1* knock-out (*n* = 13), and wild type (*n* = 10) kidney tissues were used. The procedures for quantitative gene expression analysis (qRT-PCR) are reported in the supplementary materials.

### Statistics

The associations were calculated by Spearman’s rank correlation test, while the differences in uEGF/MCP1, uEGF, and uMCP1 values between each of the five Mayo Clinic Imaging classes were assessed by the Kruskal Wallis nonparametric test. *P* values < 0.05 were considered statistically significant. We evaluated the performance of baseline ratio of uEGF/MCP1 in predicting two CKD progression risk categories (slow: Mayo Clinic Imaging Classes1A–1B; fast: Mayo Clinic Imaging Classes 1C–1E) by conditional inference tree (ctree) analysis as implemented in the “partykit” [17] package using “R statistical software”, version 3.6.3 (R Core Team 2020).

## Results

### Features of ADPKD patients

The clinical characteristics of the cohorts are shown in Table 1. In the discovery ADPKD cohort, the baseline uEGF levels correlated negatively with age (*r* = − 0.27, *p* = 0.02), serum creatinine (*r* = − 0.66, *p* < 0.001), and TKV adjusted for height (*r* = − 0.48, *p* < 0.001), and positively with eGFR (*r* = 0.63, *p* < 0.001). Conversely, the uMCP1 levels correlated positively with age (*r* = 0.27, *p* = 0.02), serum creatinine (*r* = 0.54, *p* < 0.001), and TKV adjusted for height (*r* = 0.57, *p* < 0.001), and negatively with eGFR (*r* = − 0.57, *p* < 0.001). The baseline uEGF/MCP1 ratio in the same cohort was negatively correlated with age (*r* = − 0.39, *p* < 0.001), serum creatinine (*r* = − 0.80, *p* < 0.001) and HtTKV (*r* = − 0.67, *p* < 0.001), and positively correlated with higher eGFR (*r* = 0.81, *p* < 0.001), but did not differ between sexes.

In the validation cohort, we confirmed that their uEGF levels negatively correlated with age (*r* = − 0.45, *p* < 0.001), serum creatinine (*r* = − 0.75, *p* < 0.001), and TKV adjusted for height (*r* = − 0.46, *p* < 0.001), and positively correlated with eGFR (*r* = 0.72, *p* < 0.001). Conversely, their uMCP1 levels positively correlated with serum creatinine (*r* = 0.30, *p* < 0.001) and TKV adjusted for height (*r* = 0.49, *p* < 0.001), and negatively with eGFR (*r* = − 0.40, *p* < 0.001), but did not significantly differ by age and sex. Similar to the discovery cohort, their baseline ratio of uEGF/MCP1 inversely correlated with age (*r* = − 0.36, *p* < 0.001), serum creatinine (*r* = − 0.63, *p* < 0.001), and TKV adjusted for height (*r* = − 0.60, *p* < 0.001), and positively correlated with eGFR (*r* = 0.69, *p* < 0.001).

### The uEGF/MCP1 ratio correlates with the Mayo Clinic Imaging Classes

**Fig. 1:**
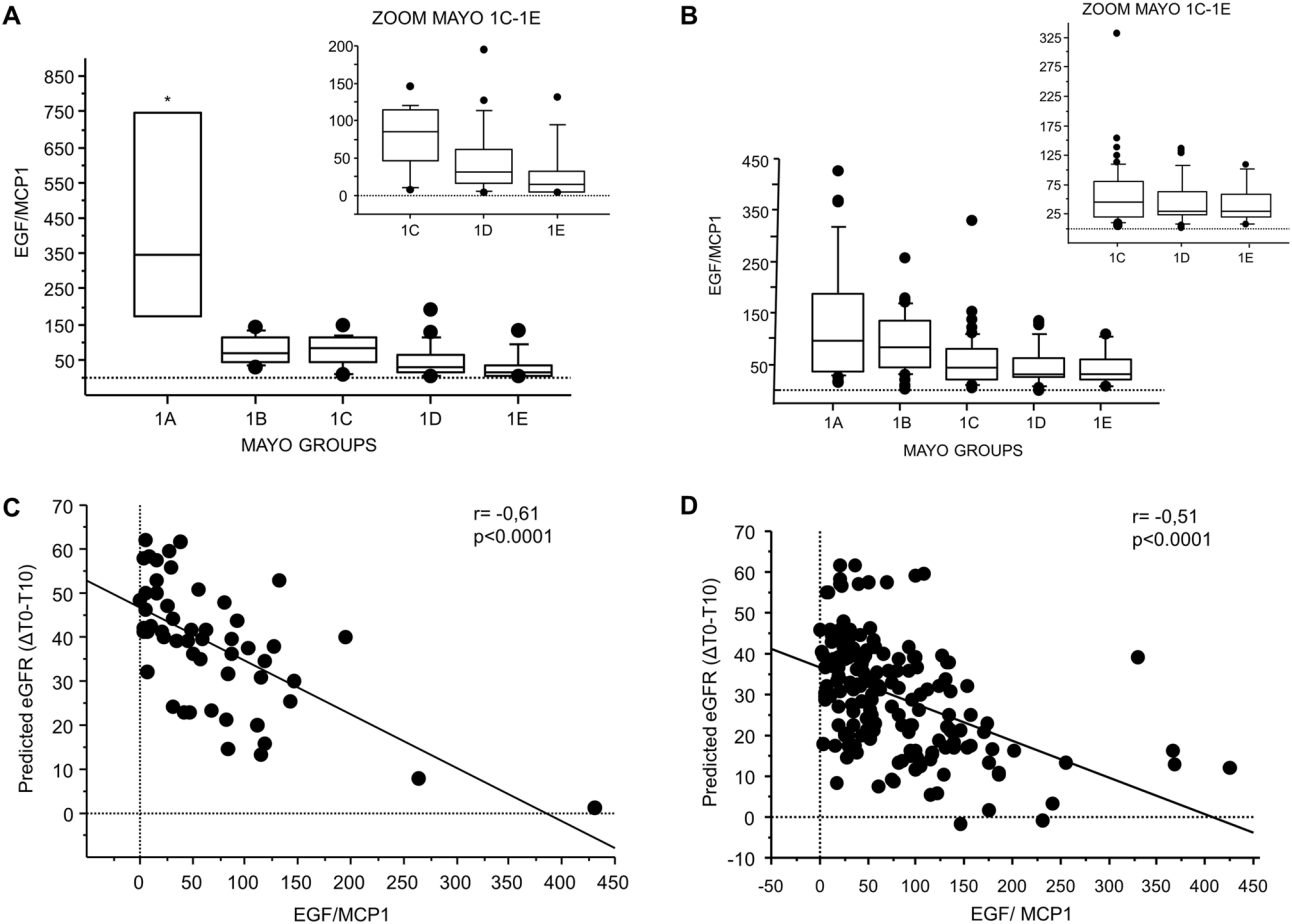
Distribution of uEGF/MCP1 across Mayo Imaging Classification classes. uEGF/MCP1 discriminates Mayo Clinic Classes in the discovery (*p* < 0.001) (**A**) and validation (*p* < 0.001) (**B**) cohorts (Kruskal Wallis). uEGF/MCP1 correlates with predicted variation of eGFR (ΔT0-T10) in the discovery cohort (**C**) and in the validation cohort (**D**) (*p* < 0.0001). Urine EGF and MCP1 values were normalized to urine creatinine (uCr) and are reported as picograms per milligram Creatinine (pg/mgCr). The ratio of urine EGF/MCP1 is reported as picogram per picogram (pg/pg)

In the discovery cohort, 63 patients with complete volume measurements were classified according to the Mayo Clinic Imaging Classification. Among them, 62 patients were typical ADPKD, in particular 6 patients belonged to class 1A, 11 to class 1B, 16 to class 1C, 20 to class 1D and 9 to class 1E. One male patient was not classified as typical ADPKD because of a previous nephrectomy and 11 patients were not classified due to missing clinical data. We found that baseline uEGF/MCP1 discriminates between Mayo Clinic Imaging Classes (Fig. 1A,  *p* < 0.001) better than uMCP1 alone (Supplemental Fig. 1, *p* = 0.02). On the contrary, uEGF failed in this purpose (Supplemental Fig. 1, *p* = 0.07), although it showed a trend of reduction towards Class 1E.

All patients from the validation cohort had typical patterns of ADPKD by magnetic resonance imaging and were classified as 1A (*n* = 30), 1B (*n* = 56), 1C (*n* = 47), 1D (*n* = 32) and 1E (*n* = 12) by Mayo Clinic Imaging Classification. Here, we confirmed that the baseline uEGF/MCP1 discriminates between different Mayo Clinic Imaging Classes (Fig. 1B , *p* < 0.001) better than each biomarker separately (Supplemental Fig. 1). Since the PROPKD score is an additional established risk predictor, we analysed the distribution of the uEGF/MCP1 ratio at baseline across the PROPKD categories (low, medium, high risk) of the discovery cohort. Unlike the Mayo Classification, this analysis was not statistically significant, although the EGF/MCP1 ratio showed a trend of reduction towards the high risk category (data not shown).

### The uEGF/MCP1 ratio correlates with the predicted variation of eGFR

We also leveraged the online tool that allows to predict the future eGFR at 10 years (T10) using the Mayo Clinic Imaging Classes, serum creatinine, age, race, gender, and eGFR at baseline (T0) (https://www.mayo.edu/research/documents/pkd-center-adpkd-classification/doc-20094754). We then calculated the predicted eGFR variation (ΔT0–T10) by subtracting the estimated eGFR from its baseline value. uEGF (*r* = − 0.36, *p* = 0.006; *r* = − 0.35, *p* < 0.0001) and uMCP1 (*r* = 0.46, *p* < 0.001, *r* = 0.44, *p* < 0.001) correlated with the ΔT0–T10 in both the discovery and validation cohort, respectively. However, uEGF/MCP1 provided better correlation with the predicted variation of eGFR in both the discovery (ΔT0–T10, *r* = − 0.61, *p* < 0.001) and validation cohort (ΔT0-T10, *r* = − 0.51, *p* < 0.001; Fig. 1C and D).

### The uEGF/MCP1 ratio as a marker of CKD progression

**Fig. 2:**
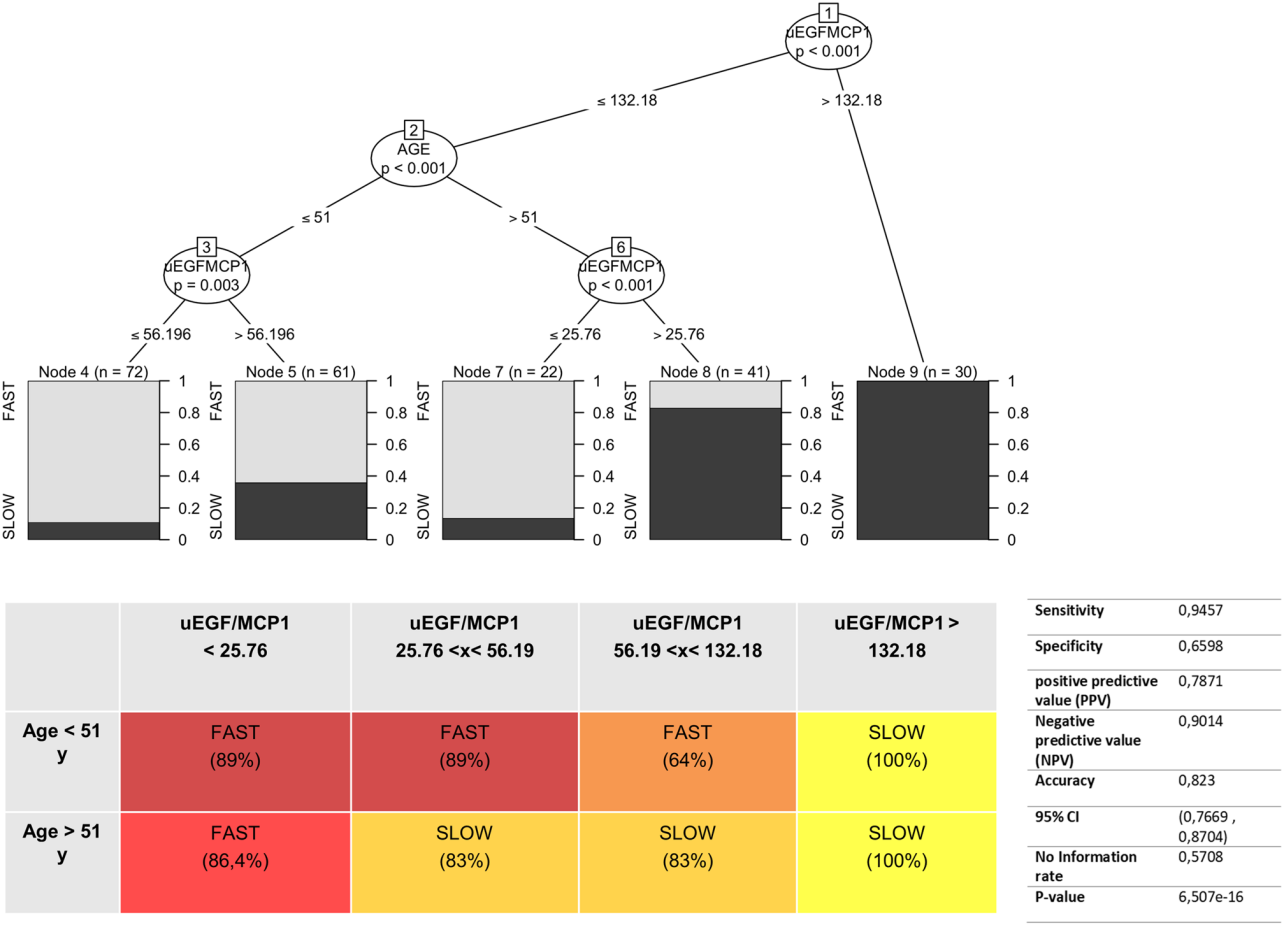
uEGF/MCP1 as biomarkers of progression. Cut-off values of uEGF/MCP1 across the risk categories (slow and fast progressor) according to age. In the table performance characteristics of the uEGF/MCP1 conditional inference tree (ctree) algorithm for the identification of slow and fast CKD progression in ADPKD are reported. Decision trees are useful tools for making predictions, which build regression models in the shape of a tree structure. Decision trees are represented as a graph that illustrates possible outcomes of different decisions according to a set of parameters. In the graph, nodes represent a decision test that focuses on a single variable and then moves to another node based on the outcome, which is indicated by the leaves of the tree

**Fig. 3:**
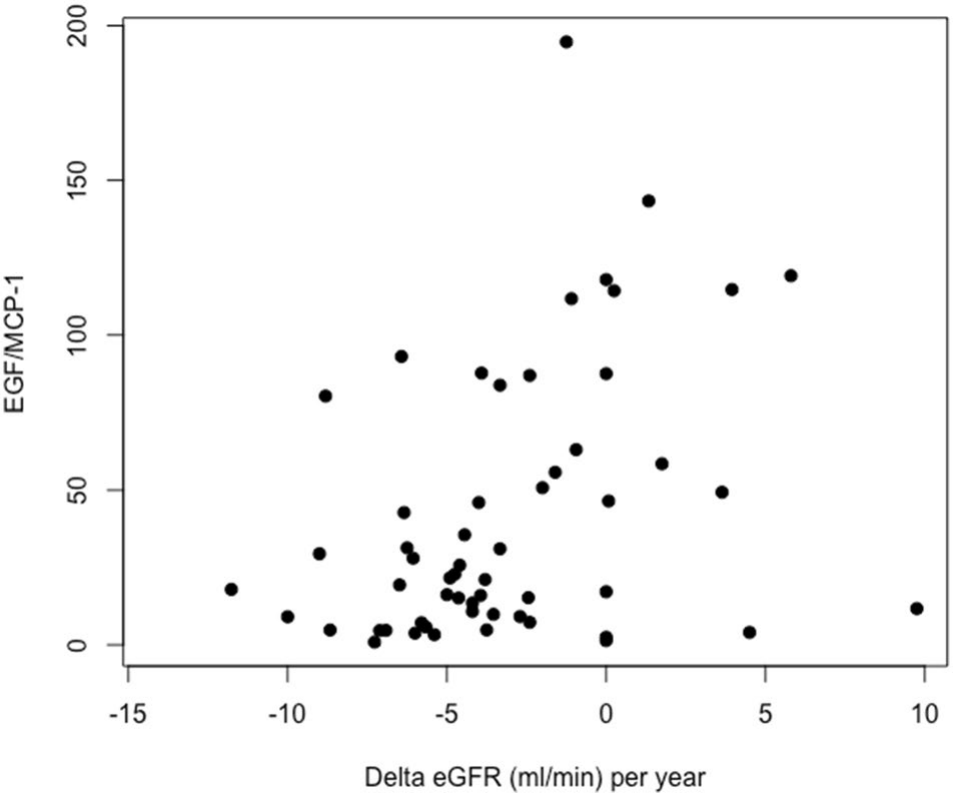
uEGF/MCP1 correlates with renal outcome (variation of eGFR: Delta-eGF*R* = eGFR_T0_-eGFR_TFU_) in the discovery cohort. T0: time of recruitment; TFU: time of follow-up

Given that uEGF/MCP1 performed well in both the discovery and validation cohorts in their correlation with the individual Mayo Clinic Imaging Classes, we combined the cohorts to maximize the sample size and evaluate the performance of our prognostic testing. We used Conditional Inference Trees for the prediction of slow (Mayo Clinic Imaging Classes 1A–1B) and fast (Mayo Clinic Imaging Classes 1C–1E) CKD progression in ADPKD, achieving cut-off levels of uEGF/MCP1 for the slow and fast progressor categories according to age. Specifically, a uEGF/MCP1 value > 132 discriminates slow progressors with 0% error (100% slow). Age should be considered for uEGF/MCP1 < 132 as follows: in patients < 51 years, a value of uEGF/MCP1 < 56.19 discriminates a fast progressor with an error of 11% (89% fast); in patients > 51 years, a uEGF/MCP1 value < 25.76 discriminates a fast progressor with an error of 13.6% (86.4% fast) (Fig. 2). Intermediate uEGF/MCP1 values identified fast progressors with slightly lower accuracy but high sensitivity (Fig. 2). Sensitivity, specificity, positive (PPV), negative predictive values (NPV) and accuracy of uEGF/MCP1 that predicts slow and fast CKD progression in ADPKD were also measured (Fig. 2). We then tested whether genetic data (matching the 3 classes of the PROPKD score: (i) *pkd*2 mutation, (ii) nontruncating *pkd*1 mutation; (iii) *pkd1* truncating mutation) and basal uEGF/MCP1 could predict renal outcome in the ADPKD discovery cohort. Linear regression analysis revealed that uEGF/MCP1 at baseline is a better predictor of renal outcome compared to genetics (Table 2). More importantly, after collecting the follow-up data for the discovery cohort we found that basal EGF/MCP1 ratio correlated with renal outcome, in terms of Delta-eGFR per year (*p* = 0.006 *r* = 0.36, Fig. 3).

**Table 2:** Performance characteristics of the combined uEGF/MCP-1 ratio and genetic mutations categorized according to the PROPKD to predict Delta-eGFR/year by linear regression analysis

Variable	Beta	Std Error	*P* Value
EGF/MCP-1	0.040230	0.008749	5.09e−05
Genetics			
PKD2 mutation	Ref.		
Nontruncating PKD1 mutation	− 2.432236	1.146757	0.0409
Truncating PKD1 mutation	0.446168	0.914918	0.6287

### Analysis and validation of EGF and MCP1 gene expression

**Fig. 4:**
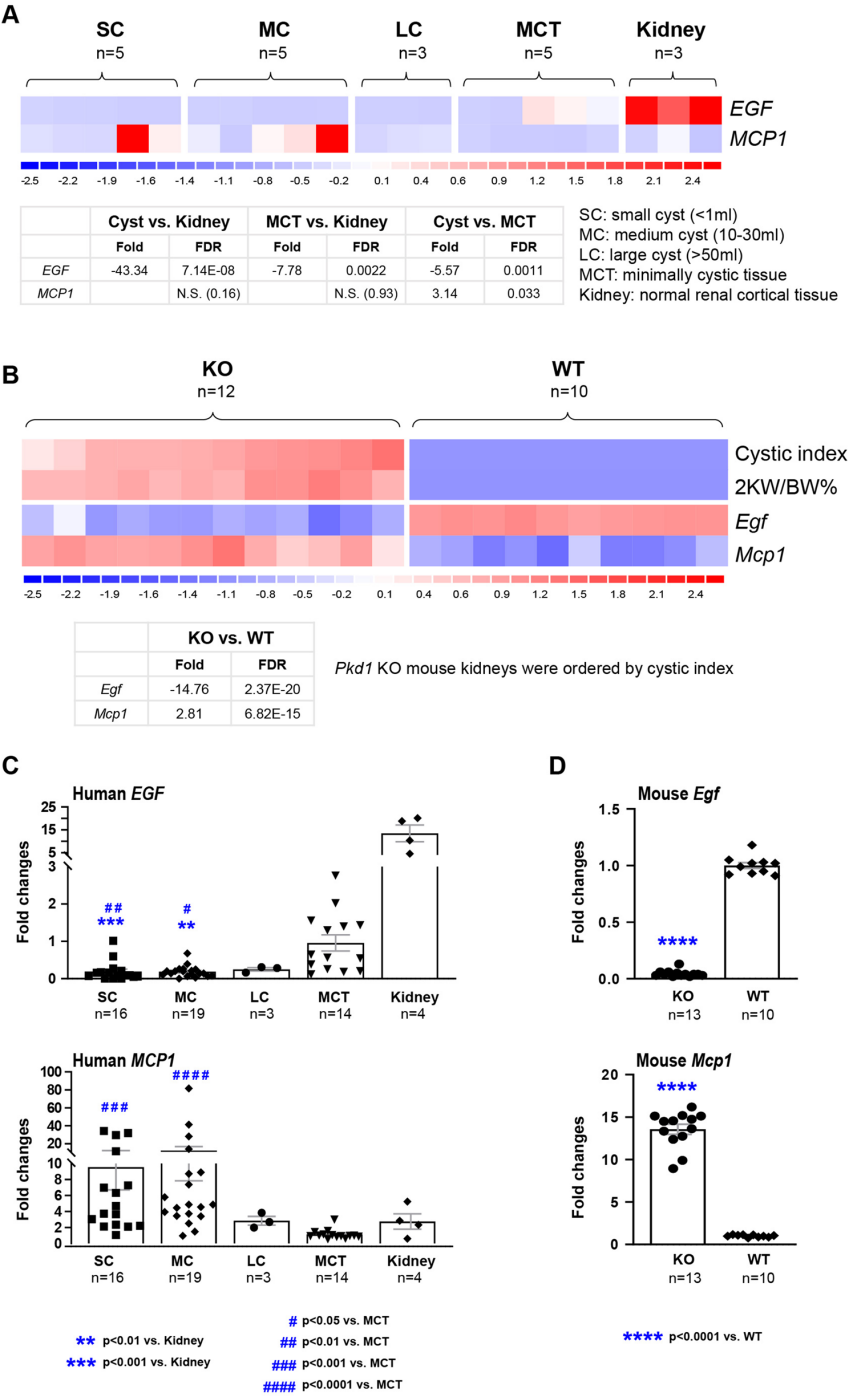
EGF and MCP1 gene expression. Gene profiling of EGF and MCP1 in human PKD1 renal cysts (**A**) and Pkd1 knock-out mouse kidneys (**B**). In the heatmap, each column represents an individual sample; each row represents the standardized gene expression values across all samples by z-score normalization (z-scores are computed on a gene-by-gene basis by subtracting the mean and then dividing by the standard deviation, and reflect the distance from the mean in units of standard deviation). Red indicates greater expression than the mean (white) value, and blue indicates less than the mean value. The colour scale under the heatmap displays the range of z-scores. **C** qRT-PCR analysis of EGF and MCP1 in an expanded set of cyst and MCT samples derived from the human PKD1 kidneys, and normal renal cortical samples. **D** qRT-PCR analysis of Egf and Mcp1 in Pkd1 knock-out and wild type kidneys. Data are expressed as mean ± SEM; statistical significance was determined using the Kruskal–Wallis nonparametric test or unpaired t test with Welch's correction

To provide further insight into the biology underpinning the variation of uEGF and uMCP1 in ADPKD patients, we analysed *EGF* and *MCP1* gene expression at the tissue level both in human *PKD1* and mouse *Pkd1* knock-out cystic tissues. The expression levels of *EGF* and *MCP1* were derived from microarray experiments on 13 renal cysts of different sizes, 5 minimally cystic tissue (MCT) from five PKD1 human polycystic kidneys and 3 normal renal cortical samples as control tissue. Gene expression showed *EGF* down-regulation (-5.6-fold, FDR = 0.001) and *MCP1* up-regulation (3.1-fold, FDR = 0.03) in PKD1 renal cysts compared to MCT (Fig. 4A). In addition, microarray analysis on *Pkd1* knock-out mouse kidneys confirmed the findings of the human expression studies showing down-regulation of *Egf* (− 14.8-fold, FD*R* = 2.37E−20) and up-regulation of *Mcp1* (2.8-fold, FDR = 6.82E−15) compared with WT kidneys (Fig. 4B). To confirm the microarray data, quantitative real-time polymerase chain reaction was used to compare the expression levels of *EGF* and *MCP1* genes in an independent set of cysts (*n* = 38: SC = 16, MC = 19, LC = 3) and MCT (*n* = 14) samples derived from human PKD1 kidneys, as well as normal renal cortical samples (*n* = 4) (Fig. 4C). Quantitative real-time polymerase chain reaction of *Egf* and *Mcp1* in Pkd1 knock-out and wild type kidneys was also performed (Fig. 4D). In both human and mouse experiments the overall fold changes by quantitative real-time polymerase chain reaction are larger than those identified by microarray analysis.

## Discussion

In this study we tested uEGF/MCP1 as a clinical biomarker for the risk of CKD progression in ADPKD. The biomarker was negatively correlated with HtTKV and concordant with the stratification of patients based on the Mayo Clinic Imaging Classification in two independent cohorts. Unlike the Mayo Clinic Imaging Classification, the uEGF/MCP1 could not discriminate across PROPKD categories, while the combination of genetic data and baseline uEGF/MCP1 ratio demonstrated that uEGF/MCP1 remained significant in predicting renal outcome in the discovery ADPKD cohort. The two cohorts were then combined to assess the prognostic performance of uEGF/MCP1. To this end, we used Conditional Inference Trees to identify slow (Mayo Clinic Imaging Classes1A–1B) and fast (Mayo Clinic Imaging Classes1C–1E) ADPKD progressors and obtained a highly sensitive model with cut-off levels of uEGF/MCP1 according to age. Although the follow-up in this study was relatively short for a slow-progressing disease like ADPKD, we found that uEGF/MCP1 at baseline correlated with renal outcome in terms of delta eGFR.

The specific trophic effect of EGF on kidney cells is well known, as its key functions in cell differentiation and regeneration allows it to modulate tissue response to injury [9, 18]. EGF tissue expression and urinary excretion decrease after kidney injury [12, 18, 19], as well as in ADPKD [12, 20] where EGF tissue expression decreases in human PKD1 cysts [10] and the growth factor receptor system, involved in tubular cell proliferation, is imbalanced, suggesting its detrimental role in ADPKD [20, 21]. Moreover, EGF mRNA levels decreased in cystic kidneys of an ADPKD murine model, while a number of growth factor genes increased with disease progression demonstrating an involvement of EGF with the progression of cystic lesions instead of ADPKD cystogenesis [22]. uEGF excretion mirrors intrarenal EGF expression and, better than serum EGF, it showed its independent predictive value of CKD progression in several large cohorts [19]. Moreover, Ju et al. demonstrated that the addition of uEGF to standard clinical parameters substantially improves the ability to predict renal outcomes in a heterogeneous CKD population [19]. We previously demonstrated the decrease of uEGF in ADPKD patients as a prognostic marker of incipient renal insufficiency [12], here we confirmed, in a larger cohort, that lower baseline uEGF predicts eGFR decline in ADPKD patients.

In contrast to EGF and growth factor expression, the upregulation of Mcp1 precedes macrophage infiltration and promotes macrophage accumulation and cyst growth in Pkd1-knock-out mouse models. In addition, the double-knock-out mouse for Pkd1 and Mcp1 showed a significantly decreased rate of cyst growth and improved kidney function suggesting a substantial role of MCP1 in macrophage-mediated cyst growth [23]. MCP1 is a potent chemotactic factor for monocytes that plays an important role in inflammatory processes. Its tissue expression was up-regulated in the kidney of a rat model of PKD and reduced by an inhibitor of MCP1 synthesis, although it did not prevent renal cyst growth [24]. The authors suggested that the MCP1 synthesis inhibitor did not completely abrogate macrophage accumulation in the renal interstitium which could still contribute to cyst growth. In humans, MCP1 expression increases in PKD1 kidneys, [10] and indeed, high levels of uMCP1 in ADPKD patients have been found [19], which correlated with kidney cyst size, and were suppressed after tolvaptan treatment [25]. In a longitudinal study, [14] uMCP1 was investigated as a marker of disease progression because its baseline excretion increased in a cohort of 55 ADPKD patients prior to a substantial increase in serum creatinine concentration or urine protein excretion. The disease progression predictive power of high levels of uMCP1 was also indicated by a study on mice with PKD where uMCP1 increased earlier than serum creatinine and blood urea nitrogen [26]. It has been reported that a hypoxic environment induced by the pressure of growing cysts stimulates the expression and release of hypoxia-inducible factor-1α and the pro-angiogenic gene vascular endothelial growth factor, which in turn, can upregulate the expression of MCP1 [27].

In an ADPKD cohort, uMCP1 correlated positively with height-adjusted TKV and negatively with the eGFR slope. Furthermore, a multivariate model including urinary levels of β2-microglobulin, MCP1 and vascular endothelial growth factor improved the ability to predict the decline of eGFR in ADPKD patients compared with height-adjusted TKV alone [27]. Likewise, baseline uMCP1, as well as the *β*-2-microglobulin, were associated with eGFR decline in the ADPKD cohort, and, when it was added to a model containing conventional risk markers that explained annual changes in eGFR its performance significantly increased [13]. According to these findings, we observed that baseline uMCP1 alone is a better predictor of eGFR decline in ADPKD compared to uEGF, though we used the urine spot test instead of 24 h-urine samples [13]. The 24-h urine sample may be more accurate than the urine spot tests used in most other studies because of the circadian rhythm in the urinary excretion of the markers, but it is inconvenient due to the long collection times, which can sometimes be inaccurate and at risk of bacterial contamination. Instead, the use of second morning urine in our study, besides being more practical, ensures the quality of the urine sample as the specimen can be collected directly in the clinic upon the patient’s arrival, thereby significantly shortening the time between sample collection and processing.

We examined whether decreased uEGF and increased uMCP1 levels in our patients mirrored changes in their gene expression in human and murine cystic tissues. Indeed, we found a significant down-regulation of *EGF* and up-regulation of *MCP1* in both human and murine PKD1 cystic kidney tissues from previously published microarray data, and confirmed this by qRT-PCR of additional cystic samples [10]. Taken together, the data suggest that uEGF and uMCP1 levels mirror changes in the cystic tissues, supporting the notion that they are pathophysiological biomarkers of ADPKD, although the exact mechanisms of EGF and MCP1 in cystogenesis have not been fully clarified.

Recent data confirmed an inverse correlation between uEGF and age [28]. These authors demonstrated the exponential decrease of serum and urinary EGF with age in healthy adults and children, emphasizing the importance of EGF in renal maturation and growth during the first years of life [28]. The age-related decrease of EGF could be ascribed to a reduced capacity of the kidney to regenerate and to recover renal function. Our data confirm a significant, negative correlation between uEGF and age, as well as for uEGF/MCP1; the central role of age in the ctree statistical model, which allows a rapid clinical use of the data, is also highlighted.

In previous studies, uEGF/MCP1 was found (i) to correlate better with renal prognosis in patients with IgA nephropathy [15]; (ii) to be independently associated with tubular atrophy and interstitial fibrosis severity in primary glomerulonephritis [23]; (iii) to predict complete remission in primary glomerulonephritis [29]; however, uEGF/MCP1 has not been tested in ADPKD. Our data showed that baseline uEGF/MCP1 is associated with the severity of disease, better than uEGF and uMCP1 alone. The stronger correlation of baseline uMCP1 levels with height-adjusted TKV and with the predicted variation of eGFR at 10 years (ΔT0–T10), compared to uEGF, may be due to the involvement of MCP1 in cyst growth, and likely, in the disease pathogenesis [8]. Based on association study data, MCP1 appeared to play a major role in ADPKD compared to EGF, which seems consistent with data reported in the literature [8, 14, 26].

Although EGF and MCP1 both correlate significantly with TKV adjusted for height, the uEGF/MCP1 shows a stronger, inverse correlation with renal volume in both the discovery and validation cohorts thus emphasizing its role in the clinical setting, not least in recommending a therapy.

The unsolved issue of CKD in ADPKD patients is the difficulty in predicting progression and the lack of specific biomarkers. Genetic analysis of PKD1 truncating mutations helps the prediction of ESRD [30], but is not always available. The PROPKD score takes into account clinical and genetic data to stratify the risk of disease progression in ADPKD patients, but it is limited to patients older than 35 years or, with regard to patients younger than 35 years old, only to those suffering from hypertension and with urologic complications [30]. Currently, height-adjusted TKV, used in combination with age and eGFR, is the best imaging biomarker to predict eGFR decline in ADPKD patients [31], to help in the selection of patients for clinical trials [32], and to guide the therapeutic approach. A recent study proposed the use of the urine-to-plasma urea ratio to predict the progression of ADPKD, together with other risk markers (TKV and PKD gene mutation). Although the combined risk score of these three risk markers predicted rapid progression of the disease better than each predictor taken alone, the laboriousness of the single risk marker measurement may still represent a limitation [33].

Some limitations of this study that might hamper data generalizability must be stated. Specifically, the relatively small number of participants that was included, which implies the lack of a validation cohort for the Conditional Inference Tree analysis. We are aware that to perform an accurate direct comparison between the prognostic performance of uEGF/MCP1 and the PROPKD score or Mayo Clinic Imaging Classification, longer follow-up and a larger cohort are needed. A novel study is ongoing to collect extended follow-up data towards a comprehensive model to test uEGF/uMCP1 with genetic and imaging data.

In conclusion, we demonstrated the role of baseline uEGF/MCP1 as a non-invasive pathophysiological biomarker that can be used with other conventional risk markers in the clinical setting for risk stratification in ADPKD patients.

## Supplementary Information

Below is the link to the electronic supplementary material.

Supplementary file1 (DOCX 123 kb)
