# Supplementary material for: Urinary epidermal growth factor/monocyte chemotactic peptide 1 ratio as non-invasive predictor of Mayo clinic imaging classes in autosomal dominant polycystic kidney disease
Source: J Nephrol. 2022 Nov 7;36(4):987–97. doi: 10.1007/s40620-022-01468-w (PMC10227134; doi:10.1007/s40620-022-01468-w)
Supplement: Supplementary file 1 — Supplementary file1 (DOCX 123 kb) [file 40620_2022_1468_MOESM1_ESM.docx]

**SupplementaRY informations**

**URINARY EPIDERMAL GROWTH FACTOR/MONOCYTE CHEMOTACTIC PEPTIDE 1 RATIO CAN PREDICT PROGRESSION IN AUTOSOMAL DOMINANT POLYCYSTIC KIDNEY DISEASE PATIENTS**

**Journal of Nephrology**

Maria Teresa Rocchetti^1^*, Francesco Pesce^2^*, Silvia Matino^2^, Giovanni Piscopo^2^, Ighli di Bari^2^, Francesco Trepiccione^3^, Giovanna Capolongo^3^, Maria Antonietta Perniola^4^, Xuewen Song^5^, Saima Khowaja^5^, Amirreza Haghighi^5^, Dorien Peters^6^, Simona Paolicelli^2^, Paola Pontrelli^2^, Giuseppe Stefano Netti^7^, Elena Ranieri^7^, Giovambattista Capasso^3,8^, Marco Moschetta^2^, York Pei^5§^, Loreto Gesualdo^2§^ (Studio PRE.MED. (MEDicina di PREcisione) Prog.n.F/050065/01-02/X32)

*These authors contributed equally to this work

^§^Co-senior authors

^1^Department of Clinical and Experimental Medicine, University of Foggia, 71122 Foggia, Italy;

^2^Department of Emergency and Organ Transplantation, Nephrology, Dialysis and Transplantation Unit, University of Bari “Aldo Moro”, Bari, Italy;

^3^Nefrologia, Dipartimento di Scienze Mediche Traslazionali, Università della Campania “Luigi Vanvitelli”, Napoli;

^4^Nefrologia e Dialisi, Presidio Ospedaliero Valle d'Itria, Martina Franca (Taranto), Italy;

^5^Division of Nephrology, University Health Network and University of Toronto, Toronto, Ontario, Canada;

^6^Department of Human Genetics, Leiden University Medical Center, Leiden, Netherlands;

^7^Clinical Pathology Unit, Center of Molecular Medicine, Department of Medical and Surgical Sciences, University of Foggia, 71122 Foggia, Italy;

^8^ Biogem Research Institute, Ariano Irpino, Italy.

Corresponding author: Loreto Gesualdo, E-mail: [loreto.gesualdo@uniba.it](mailto:loreto.gesualdo@uniba.it)

**METHODS**

***Cytokines measurements***

The intra-assay coefficients of variation were 5.0% for uMCP-1 and 2.4% for uEGF. uEGF/MCP-1 was also measured in 59 healthy volunteers age- and sex-matched. The stability of the cytokines was tested by measuring the concentrations of the cytokines in 5 urine samples of healthy subjects at the time of collection and again after 1 year of storage. We did not observe any significant differences in the concentrations between the measurements. Furthermore, to evaluate the robustness of the data, we measured the ratio of urine EGF/MCP-1 in multiple urinary samples from a random subset of patients, collected over a period of less than five days, and found a coefficient of variation of less than 5%. Samples were measured in duplicate and detected in a microplate photometer (Programmable MTP reader DV 990BV6; Gio. DeVita E C, Rome, Italy).

***Microarray validation by qRT-PCR***

Total RNA was isolated by using miRNeasy Mini Kit (Qiagen) with an on-column DNA digestion step to minimize genomic DNA contamination. cDNA was generated using High-Capacity cDNA Reverse Transcription Kit (Thermo Fisher Scientific). Real time qPCR was carried out on a ViiA 7 Real-Time PCR System (Thermo Fisher Scientific), using Power SYBR® Green PCR Master Mix (Thermo Fisher Scientific). Primer sets were designed to the exon sequences using Primer3 software (<https://bioinfo.ut.ee/primer3-0.4.0/>) (Supplemental Table 1). Absolute quantification was performed by constructing a standard curve for each primer set using serial dilutions of human or mouse genomic DNA. B2M and Hprt were identified by RefFinder^43^ as most stable reference genes in our human and mouse kidney samples, respectively and used for qPCR data normalization. qPCR results expressed as fold changes over MCT (human) or WT kidney (mouse). GraphPad Prism 9.0.1 was used for statistical analysis. Statistical significance was determined using Kruskal-Wallis nonparametric test with multiple comparisons or unpaired t test with Welch's correction.

**Supplemental Table 1.** Primer sets were designed to the exon sequences using Primer3 software (https://bioinfo.ut.ee/primer3-0.4.0/)

| **Primers used for qPCR** | | | |
| --- | --- | --- | --- |
| **Gene name** | **Primer** | **Sequence (5’→3’)** | **Amplicon** |
|  |  |  | size (bp) |
| h*EGF* | F | TGAGGAGTCGAGCAGAGATG | 78 |
|  | R | CAAGGTTGAGGGCAAGAGG |  |
| h*MCP1* | F | TCAAACTGAAGCTCGCACTC | 86 |
|  | R | GAATGAAGGTGGCTGCTATG |  |
| h*B2M** | F | GAGTGCTGTCTCCATGTTTGATGT | 71 |
|  | R | AAGTTGCCAGCCCTCCTAGAG |  |
| m*Egf* | F | ATAGATGGAATGGGCACAGG | 102 |
|  | R | AGGTCTGTAGGAGGGTAGGTG |  |
| m*Mcp1* | F | AGAGCCAGACGGGAGGAAG | 83 |
|  | R | ATGGTGGTGGAGGAAGAGAG |  |
| m*Hprt** | F | CCTAAGATGAGCGCAAGTTGAA | 86 |
|  | R | CCACAGGACTAGAACACCTGCTAA |  |
| *The housekeeping genes used for qPCR normalization | | | |

**FIGURES**

**
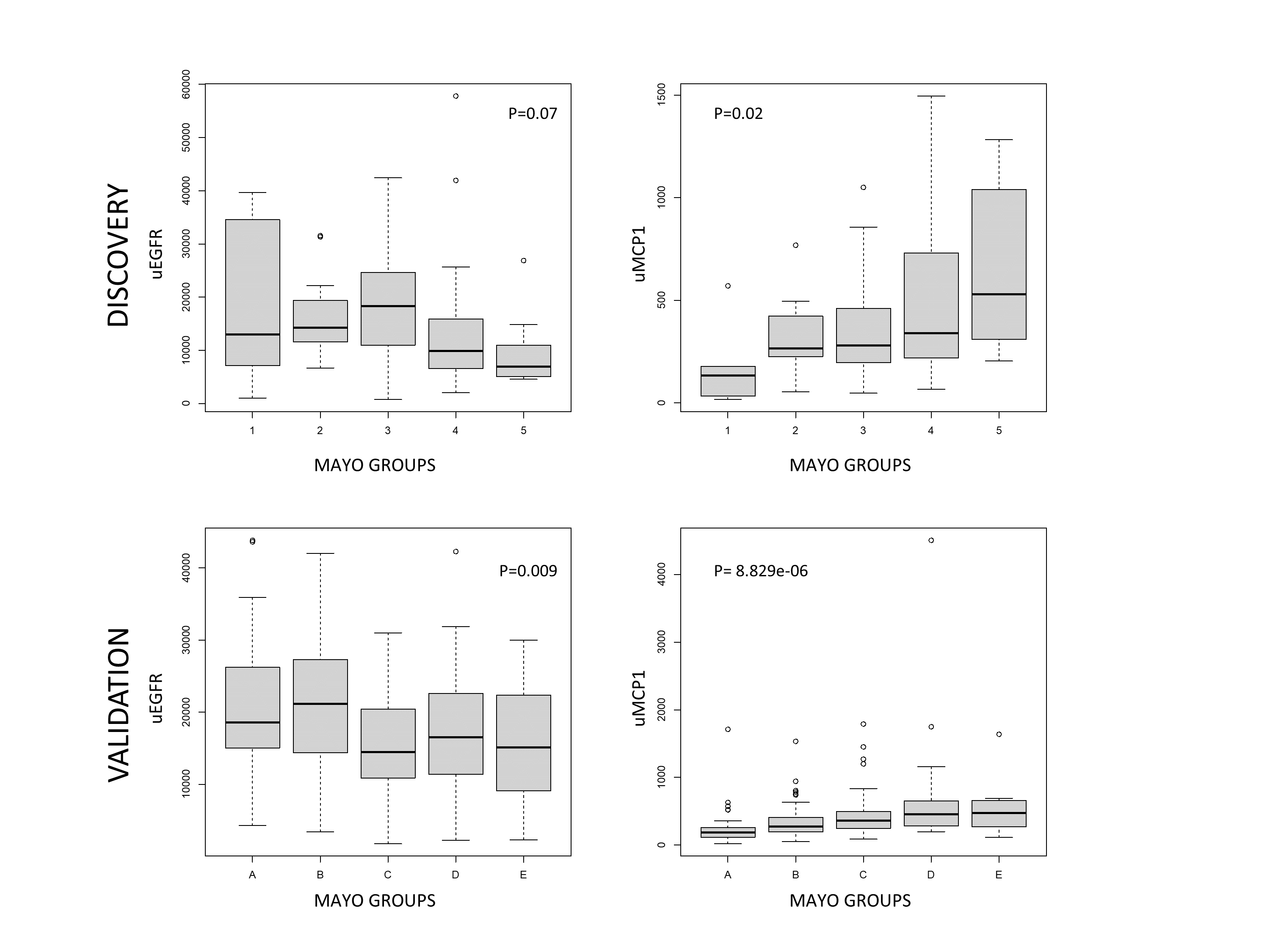
**

**Supplemental Figure 1.** Distribution of uEGF and uMCP1 across Mayo Imaging Classification classes. Baseline uEGF failed to discriminate Mayo Clinic classes in the discovery (p=0.07) left panel) while it does in validation (p=0.009) (lower left panel) cohort; baseline uMCP-1 discriminates between Mayo classes in both cohorts (p<0.01 for both, right panels) (Kruskal Wallis).
